# The dual-use problem, scientific isolationism and the division of moral labour

**DOI:** 10.1007/s40592-014-0004-9

**Published:** 2014-10-10

**Authors:** Thomas Douglas

**Affiliations:** 1 Oxford Uehiro Centre for Practical Ethics, Faculty of Philosophy, University of Oxford, Suite 8, Littlegate House, St Ebbes Street, Oxford, OX1 1PT, UK; 2 Brasenose College, University of Oxford, Oxford, UK

**Keywords:** Academic freedom, Division of moral labour, Dual-use problem, Nuclear physics, Scientific isolationism, Synthetic biology, Value of knowledge, Uncertainty

## Abstract

The dual-use problem is an ethical quandary sometimes faced by scientists and others in a position to influence the creation or dissemination of scientific knowledge. It arises when (i) an agent is considering whether to pursue some project likely to result in the creation or dissemination of scientific knowledge, (ii) that knowledge could be used in both morally desirable and morally undesirable ways, and (iii) the risk of undesirable use is sufficiently high that it is not clear that the agent may permissibly pursue the project or policy. Agents said to be faced with dual-use problems have frequently responded by appealing to a view that I call scientific isolationism. This is, roughly, the view that scientific decisions may be made without morally appraising the likely uses of the scientific knowledge whose production or dissemination is at stake. I consider whether scientific isolationism can be justified in a form that would indeed provide a way out of dual-use problems. I first argue for a presumption against a strong form of isolationism, and then examine four arguments that might be thought to override this presumption. The most promising of these arguments appeals to the idea of a division of moral labour, but I argue that even this argument can sustain at most a highly attenuated form of scientific isolationism and that this variant of isolationism has little practical import for discussions of the dual-use problem.

## The dual-use problem

Some nuclear physicists working in the first half of last century found themselves confronted with an ethical quandary. As they saw it, there were good reasons to seek the knowledge that they sought: not only was there a case for regarding that knowledge as valuable in itself, it was also plausible that the knowledge would have desirable applications, for example, in deterring warfare. However, there were reasons to refrain from seeking this knowledge too, since there was a risk that it would be misused, for example in unjustified nuclear attacks. Some physicists were sufficiently concerned about the risk of misuse that they were uncertain whether they ought to continue with their scientific work. There is, for example, evidence that several participants and potential participants in the Manhattan Project—the United States effort which lead to the development of the atomic bomb—were sufficiently concerned about the risk of misuse that they were ambivalent about whether they ought to be participating in the project.^1^

These physicists took themselves to be confronted with a quandary that has recently become known as the dual-use problem.^2^ We can understand this as the quandary arising whenever

1. An agent faces a choice whether to pursue P, where P is a policy or project such that pursuing P is likely to result in (i) the creation of new scientific knowledge, or (ii) the wider dissemination of existing scientific knowledge, and
2. The knowledge whose creation or dissemination is at stake could be used in both morally desirable and morally undesirable ways, and
3. The risk that this knowledge will be misused (that is, used in morally undesirable ways) is sufficiently serious that it is unclear whether the agent is morally permitted to pursue P.^3^

Note that although this formulation of the dual-use problem, like many other formulations in the literature, explicitly refers only to two conflicting values—morally desirable uses of knowledge, and morally undesirable uses—other considerations could potentially contribute to the problem. Whether the risk of misuse is sufficiently high that it is unclear whether the agent should pursue P may depend not only on how the risk of misuse compares to the prospect of morally desirable uses, but also, for example, on whether the knowledge will be valuable in itself, on whether the agent has made commitments to or not to pursue P, and various other factors.^4^

Some further brief clarifications may be helpful. First, the dual-use problem can, on the above formulation, arise both in relation to the creation of new scientific knowledge and the dissemination of existing scientific knowledge. Second, although this problem is often exemplified by reference to decisions faced by individual scientists, on the above formulation it can also arise for other individual agents (for example, science policymakers and journal editors) and, and, assuming collective agency is possible, for collectives (perhaps including the scientific community, national governments and society-at-large). Note also that the choice faced by the agent could be a choice about a particular project (for example, a particular scientific study) or about a general policy (for example, a choice about whether to require censorship of scientific journals or ‘classification’ of certain scientific information). The dual-use problem can thus arise at multiple levels.

It can also arise in otherwise very different areas of intellectual inquiry. The classic examples of the dual-use problem come from early and mid-twentieth century nuclear physics. However, recent ethical discussion of the dilemma has focused on the life sciences. It has been suggested that some research in molecular biology poses dual-use problems because the knowledge it produces could be used to create human pathogens or other biological agents whose intentional or negligent release into the environment would have devastating consequences. Discussion of this concern was triggered in part by developments in genome synthesis which have been taken to hold out the prospect of creating ‘designer’ pathogens or recreating historical pathogens, such as the smallpox virus or the 1918 Spanish Influenza virus, to which most people are no longer likely to be immune.^5^ Discussion has been stimulated further by two studies which resulted in the creation of variants of the H5N1 influenza virus that were transmissible by air between ferrets (and thus, perhaps, between humans).^6^

Neuroscience is another scientific area in which dual-use problems have been thought to arise (Dando 2005, 2011; Marks 2010). For example, rapid development recently in neuroimaging—particularly in functional magnetic resonance imaging—has provided new research tools for neuroscientists, and new diagnostic and prognostic tools for clinicians. But it is possible that new imaging technologies will also have applications in lie-detection. There are thus concerns that they might be used to violate privacy, perhaps as an aid to unethical interrogation practices (Wolpe et al. 2010). Neuroscientists are also beginning to understand the neural bases of various human behaviours. For example, there has been a flurry or work recently on the role of the hormone and neurotransmitter oxytocin in facilitating trust and other so-called ‘pro-social’ behaviours (e.g., Kosfeld et al. 2005; Baumgartner et al. 2008; De Dreu et al. 2011), and it has been suggested that this work could be misused, for example, by those who wish to covertly manipulate the behaviour of others (Dando 2011).

## Scientific isolationism

An agent who takes herself to be faced with a dual-use problem, or who is reasonably thought to by others to face one, plausibly bears a deliberative burden. She may not simply ignore the putative problem; she must address it in some way.

There are two obvious ways in which she might discharge this burden. First, she might attempt to resolve the problem by conducting what I will call a *use assessment*. This would involve determining the likely uses of the knowledge at stake and then determining, partly on the basis of this assessment, whether she is morally permitted to proceed. Second, she might attempt to escape the problem. That is, she may attempt to change her circumstances such that the problem no longer arises. For example, it may sometimes be possible to escape a dual-use problem by developing some new means for preventing the misuse of the knowledge whose creation or dissemination is at stake without thereby foregoing desirable uses of the knowledge. Concerns about the misuse of scientific knowledge may then no longer provide any reason to refrain from pursuing P.

Historically, however, many agents who have been confronted with dual-use problems have neither sought to resolve them, nor to escape them, nor indeed to discharge the putative deliberative obligation in any other way. Instead, they have sought to deny that any such obligation exists, maintaining that they may continue to promote scientific knowledge without resolving or escaping the problem. Often this claim is made rather obliquely. Robert Oppenheimer, the head of the Manhattan project who himself expressed ambivalence about his work said, in attempting to justify it, “[i]t is my judgment in these things that when you see something that is technically sweet, you go ahead and do it and you argue about what to do about it only after you have had your technical success”.^7^ This is ostensibly a purely descriptive claim. But given that it was intended partly to diffuse moral criticism, it is clearly meant to imply that scientists *should* or at least *may permissibly* ‘go ahead’ and argue about it later.

In other cases agents confronted with dual-use problems have come closer to flatly denying that they are under any obligation to assess the likely uses of the scientific knowledge whose creation or dissemination is at stake. Some, for instance, have sought to draw a clear distinction between science and technology, or between research and development, maintaining that questions about morally desirable and undesirable applications become relevant only in the realm of development or technology. For example, in response to the publication of a story about cruise missile technology in the science section of *Time*, the Nobel Laureate Roger Guillemin stated that “[w]hat is going on there [cruise missile development] is not science but technology and engineering…. The use, including misuse or ill use, of… knowledge is the realm of politicians, engineers and technologists”.^8^ Guillemin can plausibly be interpreted as claiming here that only in the realm of technology need one appraise the uses of scientific knowledge. The implication is that in science, use assessments can be eschewed; choices about which scientific project(s) if any to pursue or promote may be made purely on the basis of scientific considerations, for example, whether a given piece of knowledge is intrinsically or theoretically interesting.^9^ I will refer to this view as *scientific isolationism,* since it holds that decisions about the direction of science can be made in isolation from certain moral, nonscientific considerations.

If scientific isolationism is correct, then an agent reasonably supposed to be faced with a dual-use problem can simply deny that she needs to address that putative problem, since she is permitted to ignore the likely applications of a piece of knowledge in deciding whether to contribute to its production or dissemination. She is thus permitted to ignore the very factor—risk of morally undesirable uses—that is thought to generate the problem. The thought would presumably be either that how scientific knowledge will be used has no bearing on the moral permissibility of the agent’s actions, so that there is no real problem at all, or that it is has a bearing, but one that the agent is morally permitted to ignore in her deliberations.

Scientific isolationism has implications that extend beyond debates regarding the dual-use problem and the misuse of science. Indeed, the view has perhaps most frequently been invoked not in defence of science that may be used in morally *undesirable* ways, but in defence of science deemed to have no plausible morally *desirable* uses. Marie Curie famously reminded an audience at Vassar College that

We must not forget that when radium was discovered no one knew that it would prove useful in hospitals. The work was one of pure science. And this is a proof that scientific work must not be considered from the point of view of the direct usefulness of it. It must be done for itself, for the beauty of science, and then there is always the chance that a scientific discovery may become like the radium a benefit for humanity.^10^

Curie is here invoking a variant of scientific isolationism to defend the pursuit of basic science that will not clearly yield any social benefit.

In what follows, however, I will focus on the implications of scientific isolationism for scientific work that is amenable to misuse. I will consider whether it is possible to defend a form of scientific isolationism that does indeed allow agents putatively faced with dual-use problems to eschew any appraisal of the likely uses of the scientific knowledge at stake.

## Clarifications

Before proceeding to the argument proper, however, it will be necessary to offer some further preliminary remarks on scientific isolationism.

First, scientific isolationism should be distinguished from a different view with which it is frequently coupled. This is view that the state, and others outside the scientific community, ought to leave those within it a wide domain of freedom in selecting their scientific aims, and their means to achieving them (see, e.g., Bush 1945, pp. 234–235). Let us call this view scientific libertarianism.^11^ Scientific isolationism and scientific libertarianism are frequently defended together. For example, in his classic discussion, Bush (1945) moves directly from the claim that “[w]e must remove the rigid controls which we have had to impose [during World War Two], and recover freedom of inquiry”, an expression of scientific libertarianism, to the claim that “[s]cientific progress on a broad front results from the free play of free intellects, working on subjects of their own choice, in the manner dictated by their curiosity for exploration of the unknown”, arguably an expression of scientific isolationism (1945: 235). Indeed, the two views can plausibly be regarded as two aspects of the same, broader model of the relationship between science and society—a model that is often associated with the Enlightenment.^12^ According to this model, science should be autonomous from the rest of society. One statement of the view has it that “the only scientific citizens are scientists themselves” and that “for science to engage in the production of properly scientific knowledge it must live in a ‘free state’ and in a domain apart from the rest of society” (Elam and Bertilson 2002, p. 133). We could perhaps aptly characterise this model as the conjunction of scientific libertarianism—which asserts that science should be granted political autonomy from the rest of society—and scientific isolationism—which grants it a kind of moral autonomy.

Nevertheless, scientific libertarianism and scientific isolationism are distinct views, and they have different implications for dual-use problems. Scientific libertarianism might be drawn upon to resolve certain dual-use problems. Consider a case where a government is deciding whether to institute state censorship of scientific publications in a sensitive area, such as synthetic biology, and is reasonably deemed to be faced with a dual-use problem. Perhaps the government can resolve this putative problem by appealing to scientific libertarianism. If that view is correct, then clearly state censorship would be unjustified. However, though scientific libertarianism may allow agents to resolve dual-use problems in some circumstances, it will not enable the resolution of all such problems. Suppose that an individual scientist is considering whether to disseminate some item of scientific knowledge, free from any legal impediment to doing so, and takes herself to be faced with a dual-use problem. Here, scientific libertarianism will provide no guidance, for there is no question of constraining scientific freedom in this case; the issue what the scientist should do given the freedom she has been granted.

So scientific libertarianism does not allow us to resolve all dual-use problems. Scientific isolationism does, however, potentially provide a way out of all dual-use problems, for it denies that the consideration that generates such problems—the risk that scientific knowledge will be misused—needs to be considered.

But is scientific isolationism itself justified? In what follows I subject the view to critical scrutiny. I take as my initial target the following, rather strong variant of scientific isolationism:

*Full Isolationism.* For any policy or project P, moral agents considering whether to pursue P are not morally required to assess the likely uses of the scientific knowledge at stake (that is, the knowledge whose creation or dissemination is likely to be affected by the agent’s choice whether to pursue P).

This variant of isolationism is strong in two respects. First, it deems that agents may *always* permissibly ignore the use of the knowledge they produce. Second, Full Isolationism applies to all agents, regardless of their institutional role. For example, it applies to scientists, university administrators, government executives, and members of the general public. It also applies to both individual and collective agents, assuming that there are collective agents. Thus, it may apply to science funders, universities and governments.

It might seem uncharitable to take, as my target, such a strong version of scientific isolationism. However, I begin with this strong variant not because I wish to exclude weaker, and perhaps more plausible, variants; indeed I will turn to consider weaker variants of scientific isolationism in Sects. 6, 7 and 8. Rather, I begin with this strong variant because this will enable me to approach the assessment of scientific isolationism in a systematic way. My assessment proceeds in three steps. First, I set up a presumption against Full Isolationism. Second, I consider two arguments that might be thought to override this presumption and thus justify Full Isolationism. I argue that both fail. Finally, I consider whether it might be possible to defend a weaker version of scientific isolationism that can nevertheless do the work that has been asked of stronger versions. The aim of the subsequent discussion is thus not only (and indeed is not primarily) to assess Full Isolationism, but also to determine whether it is possible to defend *any* version of scientific isolationism that will allow those faced with putative dual-use problems to deny an obligation to resolve or escape those problems. I believe that this aim is one that is worth pursuing even if Full Isolationism is implausible. It would, after all, be quite consistent to hold that Full Isolationism is implausible while also suspecting that it might contain a kernel of truth, and perhaps a kernel that will be helpful to those faced with dual-use problems.

## The presumption against Full Isolationism

The argument for a presumption against Full Isolationism begins from a parallel between science and other domains. Outside of the realm of science, we generally think that, when an agent is deciding whether to facilitate the production or dissemination of a tool that is amenable to both morally desirable and undesirable uses, that agent should take into account its likely uses.

Consider the case of arms manufacturers. Most would judge that, in deciding whether to produce certain kinds of weapons, or to sell them to certain kinds of customer, arms manufacturers should consider the risk of misuse. Similarly, most would judge that in deciding whether to permit or support the production and sales of such arms, governments should take the possible misuse of those weapons into account.

It might be thought that weapons are a special case because they are so clearly amenable to misuse. But note that we would probably take a similar view about items whose potential for misuse is less obvious. For example, we would think that risk of misuse ought to be taken into account by those who manufacture and sell components that could be used to produce weapons, but which are primarily used for morally desirable or neutral purposes. And we would think that governments should consider risk of misuse in deciding how to regulate the production and sale of such components.

Similarly, consider those who manufacture and sell chemicals with legitimate uses (such as household cleaning agents) but which can also be used to manufacture unsafe and illicit drugs. Again, most would think that, in considering whether to manufacture such chemicals, and whether to sell them to certain kinds of customer, the manufacturers ought to consider the risk of misuse. Similarly, we would think that the government should take the risk of misuse into account in deciding whether and how to regulate the production and sale of such chemicals.

More generally, when tools are amenable to both morally desirable and undesirable uses, we generally judge that their likely uses should, at least in some cases, be taken into account when decisions that affect the creation or dissemination of those tools are made. We think that some rudimentary form of *use assessment* should be conducted. This, I suggest, creates a presumption in favour of the parallel view regarding scientific knowledge which is, after all, also a tool amenable to both morally desirable and undesirable uses.

It is true that those who produce tools amenable to both good and bad uses sometimes seek to deny any obligation to perform a use assessment. For example, arms manufacturers may claim that they need not consider the possible uses (including misuse) of the arms they produce and sell because *they* are not directly responsible for the misuse of those arms. Nor do they *intend* the arms to be misused.^13^ Such appeals are, however, widely regarded as unpersuasive. The facts that arms manufacturers do not intend misuse, and are not directly responsible for it, may somewhat weaken their obligation to conduct use assessments. But most of us nevertheless think that, at least in some cases, such an obligation is present.

## The noninstrumental value of knowledge

I have been arguing for a presumption against Full Isolationism. In this section and the next I consider two arguments *for* Full Isolationism that might be thought to override this presumption.

The first of these arguments appeals to the view that scientific knowledge has noninstrumental value—value that does not derive from its tendency to produce other things of value. This view has frequently been invoked by those who wish to defend their participation in the production or dissemination of knowledge that is prone to misuse (Glover 1999, p. 102), and one can see how it might function in a defence of Full Isolationism. The thought could be that pursuing a project or policy P that is likely to result in the creation or dissemination of scientific knowledge is morally permissible just in case the scientific knowledge at stake has (a sufficient degree of) noninstrumental value, so there is no need for those deciding whether to promote the creation or dissemination of knowledge to consider what instrumental value or disvalue it might have in virtue of the ways in which it will be used. Consideration of instrumental value would be superfluous. Indeed, it might be worse than superfluous; it might potentially distract those considering whether to pursue P from the more important matter of determining the likely noninstrumental value of the scientific knowledge at stake.

This argument depends on two claims. First, that knowledge is (or at least can be) noninstrumentally valuable. And second, that the noninstrumental value of a piece of knowledge is the sole determinant of whether it would be justified to pursue a project or policy likely to result in the creation or dissemination of scientific knowledge. But note that the second of these claims does not follow from the first. One could hold that knowledge is noninstrumentally valuable, but that its instrumental value is also relevant to decisions regarding whether to pursue P. Indeed, if knowledge has both instrumental and noninstrumental value, the natural position to hold would be that both types of value bear on such decisions. The view that instrumental value is irrelevant would be plausible only if the noninstrumental value of knowledge were a trump value, one that always over-rides the sorts of value or disvalue attached to good or bad applications of that knowledge—or at least a value that will have overriding force in all but extra-ordinary circumstances. But it is not clear why is should be so. Indeed, accepting that the noninstrumental value of knowledge is a trump value would have implausible implications. It would arguably imply, for example, that compared to the *status quo*, vastly greater resources should be expended on supporting science, and vastly fewer on other projects. Healthcare, education, social security, defence and so on should be supported only insofar as they are conducive to scientific progress.

## Uncertainty

A second argument for Full Isolationism would appeal to the uncertainty that will afflict any attempt to conduct what I earlier called ‘use assessments’—that is, attempts to determine the likely applications of the scientific knowledge whose creation or dissemination is likely to be affected by the decision whether to pursue P, and a determination, partly on the basis of this assessment, of whether it is morally permissible to pursue P. This uncertainty could stem from at least two sources. First, there is the problem that it will often be unclear in advance what knowledge will be created or disseminated through pursuit of P.^14^ Thus, suppose that an individual scientist is considering whether to pursue some project investigating *x*. It will presumably be somewhat predictable that this project could yield knowledge concerning *x.* But the content of that knowledge will generally not be clear in advance. If it were, there would be no need to pursue the knowledge. Similarly, it may be quite likely that the project will in fact yield knowledge about some other topic, *y.* Thus, when a scientist is faced with a question about whether to seek to produce knowledge of a certain kind, there will typically be uncertainty about what knowledge will actually be produced. (The same kind of uncertainty will not exist, or not to the same degree, in relation to the dissemination of existing knowledge.) Second, there will typically be uncertainty about how a given item of knowledge will be used. The possible applications of a given item of knowledge will often be unclear, and highly dependent on unpredictable contextual factors (such as which people acquire the knowledge).^15^

We can distinguish two different ways in which these concerns about uncertainty might figure in an argument for Full Isolationism. First, they might be invoked in support of the claim that attempting to determine the uses of scientific knowledge is always *futile.* 16 It never has any predictive value regarding how the knowledge that would in fact be created or disseminated due to pursuit of P would in fact be used. Second, they might be invoked in support of the claim that the costs of attempting to engage in such use assessments outweigh the benefits.

The first of these arguments seems difficult to sustain. Suppose scientists are considering whether to disseminate some piece of knowledge from, say, nuclear physics or synthetic biology. Suppose further that there is a clear mechanism via which the scientific knowledge might be used to produce weapons as well as clear evidence that some individuals or groups are interested in producing and using such weapons for the purposes of terrorism. We would, I think, be inclined to judge that the scientific knowledge in question is, *ceteris paribus*, more likely to be used for terrorist purposes than knowledge in relation to which we know of no similar mechanisms and motivations. It would be implausible to suggest that information about these mechanisms and motivations has *no predictive value*.^17^

At this point, a defender of Full Isolationism might retreat to the second of the two arguments mentioned above. She might concede that concerns regarding uncertainty do not render use assessments entirely futile, but claim that they do substantially diminish the payoffs from attempting to make such assessments, and substantially enough that the costs of making the assessments outweigh the benefits. This supporter of Full Isolationism might note that attempts to conduct use assessments are likely to come at considerable cost; to make these assessments well, it is likely that significant time, effort, expertise and financial resources would be required—all resources that could otherwise be devoted to other worthwhile activities. And if those use assessments would in any case be plagued by substantial uncertainty, the costs of conducting them might outweigh the benefits.

Concerns about the costliness of use assessments are likely to be particularly serious in the case of individual scientists. If individual scientists were to conduct use assessments in relation to each experiment they undertook or paper that they published, this would be highly burdensome and would significantly reduce the time available for doing scientific work. Note, however, that this concern could be significantly mitigated by outsourcing much of the necessary work to external agencies. One can imagine a situation in which scientists could consult an agency whose sole role was to provide specialist advice on how various types of scientific knowledge are likely to be used, as well, perhaps, as evaluations of how these different uses bear, morally, on the scientist. Individual scientists would then be left only with the tasks of predicting what knowledge their work might produce and deciding whether to rely on the assessments of the specialist agency. This agency could also provide assessments to policymakers, journal editors and other individual and collective agents in a position to influence the creation and dissemination of scientific knowledge.^18^

Note also that rejecting Full Isolationism does not commit one to the view that use assessments must be formed on the basis of explicit calculation and reasoning. Rejecting Full Isolationism entails accepting that use assessments should sometimes be made, but this is consistent with believing that those assessments could be made on the basis of simple heuristics (e.g. ‘research with obvious applications in warfare is, other things being equal, more likely to be used for military purposes than other research’) or even intuition, and if decisions were made in these ways it is not clear that they would be associated with substantial costs.

I have been presenting some grounds for doubting that (1) making use assessments in relation to scientific knowledge is futile, and (2) the costs of making such estimates are outweighed by the benefits. There is, moreover, some reason to suppose that these doubts are decisive. This can be seen by returning to the analogy between scientific knowledge and other tools amenable to both morally desirable and undesirable uses. The arguments concerning uncertainty that I offered above would, it seems to me, apply with equal force to certain other instances in which one agent contributes to the production or dissemination of a tool amenable to both good and bad uses. Suppose the government is considering whether to allow arms manufacturers within its jurisdiction to sell weapons to rebels in a country currently in the midst of a civil war. The complexities that typify such conflicts will likely introduce a high degree of uncertainty regarding how any weapons sold might be used, particularly if, as it seems we should, we take *long term* uses into account. Nevertheless, few would argue that, in such a situation, the government could plausibly ignore the likely uses of the arms whose sale is in question. We would not think, in such a situation, that uncertainty is sufficiently great so as to make use assessments futile, and moreover we would likely assume there to be ways of making such assessments such that their benefits will outweigh their costs. It is not obvious that the degree of uncertainty involved in use assessments in relation to scientific knowledge are substantially greater than those involved in this case—particularly if we limit ourselves to the applied sciences, where uncertainty regarding potential applications is arguably lessened. Thus, our intuition that uncertainty does not provide a decisive ground for eschewing use assessments in the arms sale case plausibly provides some evidence for the view that it does not constitute a decisive ground in the scientific knowledge case either.^19^

## Weakening scientific isolationism

I have argued that there should be a presumption against Full Isolationism, and I have been unable to identify any argument capable of overriding that presumption. But I now want to consider whether it might be possible to salvage some kernel of truth from scientific isolationism. Consider the following, weaker variant of the principle:

*Restricted Isolationism.* For any policy or project P, *individual researchers and research groups* considering whether to pursue P are not morally required to morally appraise the likely uses of the scientific knowledge at stake.

This principle differs from Full Isolationism in that it applies only to individual researchers and individual research groups. It does not apply, for example, to the scientific community as a whole, to governments, or to humanity as a whole. One might think that Oppenheimer, Guillemin, Curie, and other scientists who have asserted similar views, had no stronger variant of scientific isolationism in mind than Restricted Isolationism, for they do not explicitly include governments or other institutions within the scope of their comments. Moreover, restricting the scope of scientific isolationism in this way arguably renders it more plausible. It allows us to avail ourselves of at least two arguments that were not previously available.

## Lack of influence

The first of these arguments maintains that individual scientists and research institutions need not engage in use assessments because any action based on such assessments would be futile: individual scientists and research institutions cannot significantly alter the rate of knowledge production or dissemination. If one scientist or institution decides to abstain from producing or disseminating some piece of scientific knowledge because of the risk of misuse, this same knowledge will shortly be produced or disseminated by someone else.^20^

This argument depends on empirical claims that I am not qualified to answer; claims regarding the effect of scientists’ actions. Nevertheless, I wish to raise three doubts about the argument.

First, there is some anecdotal evidence that individual scientists *can* sometimes have a significant effect on the rate of scientific progress. Consider the case of recent research by Ron Fouchier and Yoshihiro Kawaoka’s groups demonstrating that the H5N1 virus can be rendered transmissible by air between ferrets.^21^ Following significant debate over whether papers based on these studies should be published in full, both researchers and a number of others agreed, in January 2012, to halt, for 60 days, “any research involving highly pathogenic avian influenza H5N1 viruses leading to the generation of viruses that are more transmissible in mammals” (Fouchier et al. 2012, p. 443). This voluntary moratorium in fact lasted a year (Fouchier et al. 2013). Since the moratorium was voluntary, any of the scientists who agreed to it could have defected from it at any time. In this context, the fact that each scientist chose to hold to the moratorium delayed research in this area by up to a year.^22^ Delays of this order or magnitude—months to years—could plausibly be sufficient for the development of new regulations or defensive strategies that would mitigate the risk that a piece of knowledge would be misused.

Second, it is possible that even very slight delays in the production or dissemination of knowledge could have very significant cumulative effects in the longer term. Since later discoveries build on earlier ones, it seems possible that a small delay in one area of research will have large knock on effects, leading to many more delays in other areas of research. If all of these are areas of research are prone to misuse, then the overall delaying effect on risk of misuse may be significant.

Third, even if individual scientists cannot significantly affect the rate at which scientific knowledge is produced and disseminated, they may still be morally required to conduct use assessments. This is because individual scientists may have reason to ensure that they do not become part of a collective that wrongfully enables the misuse of scientific knowledge, even if their own individual contribution to such misuse would be insignificant. Compare the ethics of contributing to climate change. It is sometimes said that the individual consumption decisions of individual people do not significantly contribute to climate change. However, even if this is so, many would argue that individuals have reasons to consider their carbon footprint, and to make consumption decisions that reduce it. A plausible explanation for this is that in making consumption decisions with a high carbon footprint, an individual becomes part of a collective which together wrongfully produces climate change.^23^ Arguably, individuals have a reason not to become a part of collective wrongdoing in this way. Similar reasoning might be applied to dual-use cases. Suppose an individual scientist creates knowledge about how to render the H5N1 virus air-transmissible that is highly liable to misuse. However, suppose that this individual scientist’s work does not significantly contribute to this risk. Still, by creating this knowledge, the scientist might become part of a collective (say, the group of scientists working on means for creating air-transmissible H5N1) that does substantially contribute to this risk. Each individual scientist might be obliged conduct a use assessment to ensure that they do not become part of collective wrongdoing in this way.

## The division of moral labour

I have suggested that the first argument for Restricted Isolationism faces three problems. I think there is, though, a more promising argument for Restricted Isolationism. This argument appeals to the idea of an efficient division of moral labour; the idea that we can sometimes achieve moral goals more efficiently by assigning different moral responsibilities to different people or institutions rather than assigning all individuals the same responsibilities. In the context of the dual-use problem, the idea would be that we can most efficiently ensure that science serves the public good by assigning responsibility for conducting use assessments to governments, expert bodies or other institutions, leaving scientists free to pursue scientific goals with the regulatory frameworks set by these other agents and institutions.

The basic idea of the division of moral labour is familiar in political philosophy from the work of John Rawls, who argues that we should accept a division of moral labour in which only institutions (or more precisely the subset of institutions that he refers to as ‘the basic structure’) should be assigned responsibility for realising distributive justice. Rawls (1993, pp. 268–269) spells out the idea as follows:

what we look for is an institutional division of labor between the basic structure and the rules applying directly to individuals and associations and to be followed by them in particular transactions. If this division of labor can be established, individuals and associations are then left free to advance their ends more effectively within the framework of the basic structure, secure in the knowledge that elsewhere in the social system the necessary corrections to preserve background justice are being made.^24^

The idea of a division of moral labour is also arguably a part of common sense moral thinking regarding a number of spheres of human activity. For example, it is a part of common sense thinking about law and medicine. It is standardly thought that lawyers operating in an adversarial legal system ought ordinarily to act in the interests of their own clients, setting aside the interests of those with whom their client is in dispute, leaving those who maintain and uphold legal institutions to ensure that the adversarial system generally yields just verdicts. Similarly, according to a widely held view of medical ethics, a doctor ought to act in the best interests of the patient she is currently treating, while institutions of healthcare resource allocation ought to constrain the treatments open to doctors in order to ensure that healthcare resources are allocated fairly and efficiently.

The division of moral labour is also arguably a feature of common sense thinking about capitalism. Economic agents in capitalist societies often inflict significant harms on one another through their competitive practices. Consider the case of a shopkeeper who drops his prices in order to force a new competitor out of his market. This action may cause significant harm to the new competitor—the sort of harm that, outside the context of capitalism, we might expect to be given significant consideration in deliberation. However, some would argue that, within capitalist systems, economic agents are morally permitted to engage in practices like aggressive price competition without considering the harm that this might cause to others. They are permitted to do this, it might be argued, because, although individual actions performed within a capitalist system may cause net harm, the system that allows individual economic agents to ignore market-based harms while a central government regulates the market to serve the common good is the economic system that, as a matter of fact, most efficiently serves the common good.

A similar argument could be offered for Restricted Isolationism. It could be argued that researchers should be free to pursue scientific objectives regardless of how the resulting knowledge will be used, because the scientific ethos that allows such aggressive pursuit of the truth within external regulatory constraints is the system of knowledge production that most efficiently serves the common good. There are at least three reasons to suppose that such a division of moral labour might be highly efficient. First, it would help to avoid the duplication of moral labour that would occur if both individual scientists and those who regulate them were to conduct use assessments in relation to the same scientific activities. Second, it would allow for responsibilities to be assigned to those best placed to discharge them. For example, responsibility for assessing the scientific merit of research could be assigned to scientists who are highly qualified to make such assessments, while responsibilities for conducting use assessments could be assigned to agencies that possess a mixture of scientific, security and ethical expertise. Third, centralisation of use assessments might allow for substantial economies of scale. For example, the institutions assigned responsibility for conducting use assessments may be able to conduct those assessments in relation to general *kinds* of scientific research thus obviating the need for use assessments to be conducted in relation to individual projects.

I have been suggesting that the idea of a division of moral labour is a widely accepted one. We have also now seen that there are reasons to think that a division of labour that assigns responsibility for conducting use assessments to regulatory institutions rather than individual scientists would aid efficiency. Nevertheless, I doubt that an appeal to the division of moral labour can sustain even the restricted version of scientific isolationism currently under consideration.

There are two difficulties. First, the division of moral labour argument that I have been outlining specifies an *ideal.* It specifies an ideal distribution of moral labour across agents and institutions—a distribution that would allow for maximal efficiency in the realisation of (certain) moral objectives. But realising this ideal requires collective action. It requires that both scientists and regulators play their part—both fulfil the responsibilities assigned to them. Suppose that one party does not play its part. For example, suppose that regulators do not regulate science so as to ensure that it serves the public good. Then it is not clear that the other group should fulfil only those responsibilities that it would be fulfil in an ideal division of moral labour, for there is no longer any hope that the ideal will be realised. For example, if regulators are *not* fulfilling their responsibilities, then there is no longer any hope of distributing moral labour so as to realise the economies of scale that would attached to a centralised use-assessment process. Nor is there any possibility of distributing moral labour such that use assessments are performed by those best qualified to perform them, since those bodies are, we are assuming, not fulfilling their responsibilities.

These thoughts are relevant since it is plausible that scientific governance bodies are *not* currently conducting the use assessments that they would conduct in a maximally efficient division of moral labour. Some steps have been taken in recent years to introduce a form of centralised use assessment for certain kinds of scientific research, particularly in relation to the dissemination of scientific knowledge. For example, the United States’ National Science Advisory Board for Biosecurity has taken on an advisory role in relation to the publication of microbiological research deemed to be at risk of misuse. However, for many potential dual-use problems, there is no provision for use assessments to be made by the institutions of scientific governance. For instance, though most developed jurisdictions operate comprehensive systems for the ethical oversight of human subject research, the institutions which carry out ethical review (for example, Institutional Review Boards and Research Ethics Committees) are not generally instructed to consider the risk that knowledge produced by scientific research will be misused. Their focus is on the *means* by which the scientific work will be conducted. Moreover, research that does not involve human subjects is not typically subjected to ethical review, though it may, of course, pose dual-use problems. Similarly, though many research funders take into account the likely desirable uses of scientific knowledge when making decisions about which scientific projects to fund, most do not consider the risk that the knowledge will be used in undesirable ways.^25^ These gaps in existing institutional arrangements for preventing the misuse of scientific knowledge might lead one to conclude that, even if the optimally efficient division of moral labour would have use assessments conducted by governance bodies rather than individual scientists, this is a division of labour that scientists cannot currently realise, since governance bodies are not playing their part.^26^

A second difficulty is that it is doubtful whether the optimally efficient division of labour would lead leave scientists with *no* obligation to conduct use assessments. Compare two possible divisions of moral labour. In the first, scientists need not ever conduct use assessments in relation to their work. Institutions of scientific governance are the only bodies assigned responsibility for conducting use assessments. In the second division of labour, most use assessments are conducted by governance bodies, but individual scientists and research groups must conduct at least cursory use assessments in certain cases—cases where there is a clear and severe risk that the scientific knowledge they plan to produce or disseminate will be used in highly undesirable ways, and this despite the optimal preventative policies having been adopted by governance bodies. It is difficult to see how this latter division of moral labour could be less efficient than the former. For the responsibilities placed on scientists and research groups in the latter division of labour would not, overall, be burdensome—only a cursory use assessment is required, and it will be required only in rare cases—yet it is plausible that the latter division of moral labour could prevent some seriously undesirable uses of scientific knowledge. Compared to the second, more qualified, division of labour, the first, more extreme division might seem to leave society dangerously exposed to the misuse of science for the sake of a minor reduction of the deliberative burdens on scientists.

These thoughts suggest that, at the very most, the idea of the division of moral labour will support an even further weakened version of scientific isolationism according to which *in the majority of cases*, scientists faced with choices about whether to promote the creation or dissemination of scientific knowledge need not consider how the resulting knowledge is likely to be misused, but according to which in rare cases—for example, where there is a clear and severe risk of misuse—at least a cursory use assessment must be performed.

Notice, however, that this further-weakened version of scientific isolationism will be of little use to those who wish to invoke scientific isolationism in order to avoid an obligation to resolve dual-use problems. This is because most agree that dual-use problems normally only arise in cases were there *is* a clear and severe risk of a highly undesirable use of knowledge. It is normally thought that only in these cases is there a serious question about whether it would be permissible to pursue a project or policy that can be expected to result in the creation or dissemination of that knowledge. But these are precisely the cases in which the present, further-weakened version of scientific isolationism will provide no means of escape. For this version of scientific isolationism accepts that, in *these* circumstances, scientists *ought* to conduct use assessments.

## Conclusion

Not having been able to identify any good argument for either Full or Restricted Isolationism, I tentatively conclude that we should accept neither. I think we should accept, that is, that there are cases in which agents reasonably thought to be faced with dual-use problems—including individual scientists—should morally appraise the likely uses of the scientific knowledge at stake. The most we can salvage from scientific isolationism is, I think, the idea, suggested by the division of moral labour argument, that individual researchers and research groups may permissibly eschew any moral appraisal of the likely uses of the knowledge they produce or disseminate *in most cases*. However, as we have seen, this variant of scientific isolationism has little dialectic force as a means of avoiding the need to resolve dual-use problems. Moreover, the argument for this view relies on the assumption that governance bodies are playing their part in the realisation of the optimally efficient division of moral labour, and this could be questioned.

## Footnotes

1 See, for discussion, Glover (1999, pp. 92–93), Bostrom (2007), and Lakoff (1980).

2 The quandary is also sometimes known as the dual-use *dilemma*, but I prefer dual-use *problem* since it is not clear that there is a genuine moral dilemma—in the sense of a choice structure in which the agent cannot but act wrongly—in these cases. I present the dual-use problem here as a problem that arises in relation to the creation or dissemination of *scientific knowledge*, but others have also used the term to refer to the analogous quandary that can arise in the creation or dissemination of *technologies*. Note that the term ‘dual-use’ has also been used, in a different, non-normative sense, to refer to the fact that knowledge or technologies developed for military purposes may have unforeseen civilian benefits. See, for an example of this usage, King (2005). For discussion of the term, see Selgelid (2013).

3 I take the seriousness of the risk of misuse to be a function of both the moral undesirability of the use and the likelihood with which the use will occur.

4 For discussion of a number of other values that could be relevant to dual-use problems, see Buchanan and Kelley (2011).

5 For examples of scientific developments that have driven this discussion, see Jackson et al. (2001), who report the accidental creation of a vaccine-resistent and highly lethal strain of the mousepox virus; Cello et al. (2002), who report the synthesis of a complete poliovirus genome using chemicals purchased over the internet, and based on genomic information also freely available on the net; and Tumpey et al. (2005), who report the re-creation, also through synthetic techniques, of the 1918 Spanish influenza virus.

6 For concise summaries of the studies and ensuing debate, see Evans (2013) and Vogel (2014).

7 Quoted in Jungk (1970, p. 266).

8 Roger Guillemin, in a letter to the editor, Time (September 3, 1979), p. 5. Quoted in Lakoff (1980, p. 101). See, for discussion, Glover (1999, p. 102).

9 See, for discussion, Daniels (1967).

10 Marie Curie in a Lecture at Vassar College, May 14, 1921. Quoted in Bostrom (2007, p. 145). Note that Curie’s claim here is in fact significantly stronger than *scientific isolationism*, as I defined that view above. She claims not social utility may be ignored, but that it ‘must’. However, since must implies may, we can take her to be implicitly also defending scientific isolationism as well.

11 In Hohfeldian terms, scientific libertarianism ascribes to members of the scientific community a moral claim-right to freedom from external interference in their scientific decision-making. Scientific isolationism, by contrast, assigns scientists (and others) a moral permission; a permission to eschew any moral appraisal of the likely uses of scientific knowledge.

12 This is the term given to it by Bostrom (2007). Arguably this conception of science gained the ascendancy much later than the Enlightenment. It has been argued that, in the US, it came to the fore only in the 1870s. See, for discussion, Daniels (1967).

13 Arms manufacturers could appeal here to the Doctrine of Double Effect. For a discussion of double-effect reasoning in relation to the dual-use problem, see Uniacke (2013).

14 See, for a classic statement of this view, Bush (1945, p. 241).

15 There may also be *moral* uncertainty. Even if we could fully specify a likely use of the knowledge, there might be uncertainty about its moral (un)desirability, and about how its moral (un)desirability bears on the moral permissibility of pursuing P. I leave aside moral uncertainty here, since it is not normally thought a sufficient ground for eschewing deliberation in the face of putative moral quandaries. But see Briggle (2014) for a detailed discussion of the ways in which moral uncertainty has been neglected in recent discussions of the social impacts of science.

16 Microbiologist Vincent Racaniello defended this view forcefully in a debate over the recent H5N1 ferret studies, claiming that “[f]or much of science you can't do a risk–benefit analysis. If you think you can, you're wrong” (Quoted in Roos 2012). Michael Polanyi (2000, p. 9) puts the point more generally, holding that “the aspiration of guiding the progress of science into socially beneficent channels [is] impossible and nonsensical”, and then (p. 10): “You can kill or mutilate the advance of science, you cannot shape it. For it can advance only by essentially unpredictable steps, pursuing problems of its own, and the practical benefits of these advances will be incidental and hence doubly unpredictable”.

17 It might be argued that, in order for a piece of scientific knowledge to have such clear military applications, it would have to be the result of highly *applied* scientific work, and by the time the science on a given topic has proceeded to such an applied stage, there may seem to be little hope of preventing further scientific progress on the topic in question; the field may already have acquired significant momentum. Thus, those in a position to support or impede scientific work may be faced with a variant of the Collingridge dilemma (see Collingridge 1980): either they act before the scientific work has reached an applied stage, in which case they may be able to influence whether further scientific knowledge is produced, but they have no predictive ability, or they act at an applied stage, where they have some predictive ability, but no ability to influence the production of scientific knowledge. However, it seems to me doubtful that there are many cases in which *nothing* can be done to hold back the production of a piece of scientific knowledge. See Sect. 7 below for some considerations in support of these doubts.

18 The US National Science Foundation’s ‘Science of Science and Innovation Programme’ is an example of a recent attempt to develop expert advice on the likely social effects of science. For a critical discussion, see Briggle (2014).

19 For a similar argument, see Bostrom (2007).

20 See, for example, Vandermolen (2006). For discussion, see Bostrom (2007, p. 11).

21 See, for discussion, Evans (2013) and Vogel (2014).

22 Similar points hold in relation to the moratorium on recombinant DNA research agreed by a large group of American molecular biologists in mid-1974. This moratorium lasted until the now-famous Asilomar Conference, which took place in February 1925 and resulted in a broad-based agreement on guidelines regarding the conditions under which recombinant DNA research ought to proceed—guidelines which were themselves widely respected. For a brief overview of this case, see Berg (2008).

23 For a sophisticated treatment of this approach to climate change and other similar problems, see Kutz (2007).

24 For subsequent influential discussions of the division of moral labour in political philosophy, see Nagel (1995), Murphy (1999) and Scheffler (2005).

25 Though a number of United Kingdom funding bodies did recently take steps in the direction of taking risk of misuse of knowledge into account. These funding bodies now require grant applicants to declare the risks of misuse associated with their proposed work. See BBSRC et al. (2005).

26 An analogue of this argument might be fairly widely accepted in relation to capitalism. I mentioned above a common sense division of moral labour view according to which individual economic agents should pursue their own self-interest in their economic transactions, while governance bodies ensure that market system serves the public good. However, there are certain areas in which governance bodies are plausibly failing to fulfil the responsibilities that they would be assigned in this division of moral labour. For example, governance bodies are arguably not effectively protecting the environment and animals against harm from capitalism, and for this reason, many would argue that, even if in an ideal capitalist system individual agents would not need to consider the effects of their economic transactions on the environment and animals, in actual economic systems, they are required to do so.
